# Color and Attractant Preferences of the Black Fig Fly, *Silba adipata*: Implications for Monitoring and Mass Trapping of This Invasive Pest

**DOI:** 10.3390/insects16070732

**Published:** 2025-07-17

**Authors:** Ricardo Díaz-del-Castillo, Guadalupe Córdova-García, Diana Pérez-Staples, Andrea Birke, Trevor Williams, Rodrigo Lasa

**Affiliations:** 1 Instituto de Ecología AC (INECOL), Xalapa 91073, Veracruz, Mexico; ricardo.diaz@posgrado.ecologia.edu.mx (R.D.-d.-C.); andrea.birke@inecol.mx (A.B.); 2 Instituto de Investigaciones en Inteligencia Artificial (IIIA), Universidad Veracruzana, Xalapa 91097, Veracruz, Mexico; azolla29@gmail.com; 3 Instituto de Biotechnological y Ecología Aplicada, Universidad Veracruzana, Xalapa 91090, Veracruz, Mexico; diperez@uv.mx

**Keywords:** ammonium salts, food-based attractants, chromatic response, *Neosilba*, *Lonchaea*, Lonchaeidae, trap selectivity, Mexico

## Abstract

The black fig fly, *Silba adipata* (Diptera: Lonchaeidae), is an invasive pest recently introduced to Mexico, where it has rapidly spread across fig-producing regions. Despite its economic importance, effective monitoring strategies remain poorly studied. The present study evaluated the response of *S. adipata* adults to visual (color) and olfactory (attractant) cues under laboratory and field conditions in fig orchards. No significant color preferences were observed in laboratory choice tests using nine colors or in field trials using traps of four different colors. In the laboratory, traps containing 2% ammonium sulfate solution, torula yeast + borax, or Captor + borax, captured similar numbers of flies, whereas CeraTrap^®^ was less attractive. Traps containing 2% ammonium sulfate were more effective than 2% ammonium acetate, though attraction was comparable when ammonium acetate was diluted to 0.2% or 0.02%. In the field, torula yeast + borax and 2% ammonium sulfate mixed with fig latex outperformed the 2% ammonium sulfate solution alone, although seasonal variation influenced trap performance. A high proportion of field-captured females were sexually immature. Torula yeast + borax attracted high numbers of non-target insects and other lonchaeid species, which reduced its specificity. In contrast, traps containing fig latex mixtures showed higher selectivity, although some *S. adipata* adults could not be sexed due to specimen degradation. These findings highlight the value of torula yeast pellets and 2% ammonium sulfate plus fig latex for monitoring this pest, but merit validation in field studies performed over the entire crop cycle across both wet and dry seasons. Future studies should evaluate other proteins, ammonium salt combinations and fig latex volatiles in order to develop effective and selective monitoring or mass trapping tools targeted at this invasive pest.

## Simple Summary

The black fig fly, *Silba adipata*, has recently invaded South Africa, California, USA and Mexico, where it is causing major loses in fig crop production. There are no established monitoring strategies for this pest. In the present study, we observed that flies of both sexes showed no clear preference for traps of different colors in laboratory cage tests or in traps placed in fig orchards. Testing of flies in laboratory cages indicated high attraction to the 2% ammonium sulfate solution and torula yeast + borax. Traps containing 2% ammonium sulfate solution were as attractive as 0.2% or 0.02% ammonium acetate solution, but more attractive than 2% ammonium acetate solution. Trials performed in fig orchards revealed that fly captures in traps containing 2% ammonium sulfate solution were increased if traps also contained small quantities of fig latex, although seasonal differences in fly captures were also observed. Many of the female flies captured were not sexually mature and may have been seeking sources of protein for their sexual development. Traps containing torula yeast + borax were less selective and captured higher numbers of non-target insects (mostly other flies) than traps containing 2% ammonium sulfate and fig latex. Future studies should evaluate fly attraction of these substances over crop cycle and seasons and also evaluate other ammonium compounds and attraction to volatile substances released from fig latex to develop additional tools to monitor populations of this invasive pest.

## 1. Introduction

The black fig fly, *Silba adipata* (Diptera: Lonchaeidae), is a frugivorous invasive pest that poses a significant threat to fig (*Ficus carica* L.) cultivation. Native to the Middle East and the Mediterranean region, this pest has now been reported in South Africa, California USA, and Mexico [1,2,3], far beyond its original range. In Mexico, it was first detected in the state of Morelos and subsequently in other fig-producing regions of the country [4,5,6,7]. This fly is a monophagous species with approximately six generations occurring primarily between March and November of each year [7,8]. Females oviposit in small clusters beneath the scales of the ostiole in immature syconia, which are usually smaller than 30 mm in diameter [6,9]. The larvae penetrate through the ostiole and feed internally, accelerating fruit ripening and resulting in premature fruit drop. In the ‘Brown Turkey’ fig variety cultivated in the state of Veracruz, Mexico, losses caused by *S. adipata* have been reported to exceed 60% prior to harvest [7].

Pest detection, population monitoring, and understanding the spatial and temporal distribution of pests in a crop are essential aspects of the information required for implementing effective integrated pest management (IPM) tools [10]. The sensitivity and consistency of pest responses to trap attractants, as well as the selectivity of those attractants with respect to non-target insects, are critical for the effective application of monitoring and management protocols [11]. In addition to their use for monitoring, traps and attractants can also be employed to control the insect through mass trapping, a technique that has proven effective against tephritid pests [12,13].

To date, few studies have investigated the population dynamics of *Silba adipata* in Mexico, and efficient trapping tools for growers are lacking. In contrast, numerous studies on tephritid flies have led to the development and optimization of traps and attractants [14], and have provided comprehensive guidelines for the establishment and maintenance of trapping networks for monitoring purposes and the creation of pest-free areas [15]. Among the most important factors influencing trap performance are the type of attractant and physical trap features such as the color, shape, and size, among others. Color has been the most frequently used cue in trap development and is considered to be a key characteristic in the visual attraction of tephritid flies, particularly when traps are designed to mimic host fruits [14]. Most attractants for tephritids are protein-based and rely on the premise that proteinaceous compounds are essential for female sexual development [16,17,18], and also enhance male sexual performance [19,20]. Consequently, traps baited with liquid hydrolyzed protein or torula yeast pellets are commonly used by growers to monitor tephritid pests [14,21]. The sexual maturation of *S. adipata* females also depends on the ingestion of hydrolyzed protein [22], and the Mexican phytosanitary authority SENASICA currently recommends the use of torula yeast pellets in traps targeted at *S. adipata* [5]. However, no comparative data is currently available on the response of this pest to different attractants or colors in Mexico or elsewhere.

Ammonium salts, particularly ammonium acetate [23], have also been used in tephritid monitoring, as these flies are attracted to ammonia gas released during protein decomposition [21,24,25,26]. In Europe, ammonium sulfate (2%) and hexanol, used either individually or in combination, have proven effective for monitoring *S. adipata* [8,27]. Interestingly, a combination of ammonium sulfate and hexanol was reported to capture three times as many *S. adipata* adults as either attractant alone in Greece [27]. However, the same compounds failed to capture *S. adipata* adults either individually or in combination in Mexico [28,29].

Laboratory and field observations have revealed that *S. adipata* is highly attracted to fig latex, although the reason for this attraction is still unclear [8,22,30]. In Turkey, the addition of fig latex to a mixture of ammonium sulfate and hexanol, resulted in a significant increase in the capture of *S. adipata* adults over a period of several weeks compared with the same mixture without fig latex [31]. However, no other experiments have compared different trap colors and a range of concentrations of ammonium salts, with torula yeast pellets and the contribution of fig latex in a systematic manner as tested in the present study.

The infestation of *S. adipata* in Mexico is associated with other genera of lonchaeid flies, including *Neosilba* and *Lonchaea* [7,29]. Given the high morphological and behavioral similarities among species within this family, the selectivity of attractants for monitoring should be evaluated not only in terms of non-target insects but also in relation to other lonchaeid species, in order to minimize false positives reported by growers.

The main objectives of this study were therefore (1) to evaluate the influence of color stimuli on *S. adipata* attraction to traps under both laboratory and fig orchard conditions, and (2) to compare different protein-based attractants and ammonium acetate with a reference ammonium sulfate attractant, with and without fig latex, in relation to the ammonia emissions of the attractants. Finally, four selected attractants were evaluated under field conditions based on their effectiveness in trapping *S. adipata* adults. The ability of these attractants to capture adults of *S. adipata*, as well as their selectivity in avoiding non-target lonchaeids and other insect captures, was also assessed.

## 2. Materials and Methods

### 2.1. Insects, Attractants, and Fig Orchards

Adults of *S. adipata* used in laboratory experiments were obtained from infested figs, Brown Turkey variety, collected in late October 2023 and from April to July 2024 in plantations adjacent to the villages of Tatatila (19°41′27″ N; 97°6′38″ W, 2046 m elevation) and Tenexpanoya (19°39′38″ N; 97°8′35″ W, 1840 m elevation), in the state of Veracruz, Mexico. Figs were collected from trees or as fallen fruit provided that no signs of decomposition were present. Collected figs were placed in open plastic containers (30 × 20 × 15 cm) with a 5 mm layer of vermiculite placed over a paper towel for pupation. Each container was placed in a cage (60 × 60 × 90 cm) of mosquito netting on a plastic frame and adult flies that emerged were collected from cages daily and identified as *S. adipata* by their morphological characteristics [32]. Groups of 10 to 15 flies were transferred to plastic cages (3000 cm^3^ capacity) with a source of refined sugar (Chedraui, Veracruz, Mexico), hydrolyzed yeast (MP Biomedicals, Solon, OH, USA), and a water source and maintained in a controlled temperature laboratory at 24 ± 1 °C, 65 ± 10% relative humidity, with a 12 h:12 h photoperiod (9W LED strip lights of 3500–4500 lux) (model Q09-73D, QOP Iluminación, Mexico City, Mexico) prior to their use in experiments. These same conditions were used for all the laboratory experiments described in the following sections.

Several attractants were tested under laboratory conditions and in fig orchards: (i) ammonium sulfate (99.2%, Faga Lab, Sinaloa, Mexico), a salt previously used as an attractant for *S. adipata* [8]; (ii) ammonium acetate (98.1%, J.T. Baker, Mexico City, Mexico), another salt commonly used for monitoring tephritid pests [23]; (iii) Captor 300 (Promotora Agropecuaria Universal, Mexico City, Mexico), a commercial chemically hydrolyzed plant protein, commonly used in Mexico to trap tephritid flies, which is prepared with 10 mL Captor 300, 5 g borax (J.T. Baker, Mexico City, Mexico) and 235 mL of water as stipulated in the Mexican phytosanitary guidelines for fruit fly lures [33]; (iv) torula yeast pellets (Better World Manufacturing Mexico, Mexico City, Mexico), an autolyzed torula yeast powder (45%) mixed with borax (55%) that is used at a rate of 1 pellet (5 g) per 100 mL of water [34], which is currently the attractant recommended for *S. adipata* by the Mexican government’s phytosanitary authority [5]; (v) CeraTrap (Bioibérica, Barcelona, Spain) an enzymatic hydrolyzed protein of animal origin which is highly attractive to tephritid flies and is used without dilution following the manufacturer’s instructions.

**Figure 1:**
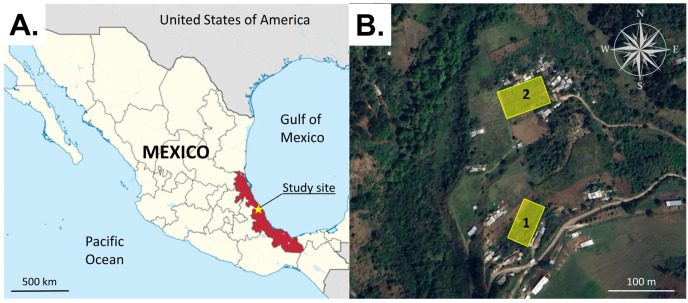
Location of the study site (yellow star) in Veracruz State (red fill) on the Gulf coast of Mexico (**A**). The study orchards 1 and 2 (yellow rectangles) were located close to the villages of Tatatila and Tenexpanoya at a distance of ~300 m from one another (**B**). Image (**A**) was adapted from https://commons.wikimedia.org/wiki/File:Veracruz_in_Mexico_(location_map_scheme).svg under the Creative Commons Attribution (CC BY-SA 3.0) (accessed on 15 July 2025). Image (**B**) was adapted from Google Earth (image date 1 April 2023), used under Google’s policies for non-commercial academic use.

The field experiments were performed during the spring–summer period when *S. adipata* adults are active in the field [7]. The duration of each experiment was determined by the number of treatments tested and the need to rotate traps within each block to obtain statistically robust samples of fly captures. Field experiments were performed in two fig orchards adjacent to the village of Tenexpanoya, Veracruz State (Figure 1A,B). Fig is considered the most important crop of this region, although other fruit trees are also cultivated, such as apple, pear, peach, avocado, and walnut, surrounded by natural forest vegetation. The area is characterized by a temperate climate (mean 20 °C) with a rainy season between June and October. The orchards were named orchard 1 (19°39′33″ N; 97°8′37″ W, 1835 m elevation) and orchard 2 (19°39′38″ N; 97°8′35″ W, 1840 m elevation) and were separated by a distance of ~300 m (Figure 1B). Each orchard comprised an area of 2500–2700 m^2^ with figs planted at intervals of 2 m, with 4–6 main branches and a height of 2.5–3.0 m. No phytosanitary measures or fertilizer treatments were applied to the crops in 2023 or during our experiments in 2024 and 2025.

### 2.2. Visual Attraction to Different Colors: Laboratory Experiment

A choice experiment was conducted using small water pan traps constructed from transparent plastic Petri dishes (90 mm in diameter), each with a circle of colored adhesive plastic (Lustrin, IKW, Monterrey, Mexico) of the same diameter that adhered to the base of the dish. The colors tested were red, orange, yellow, green, blue, violet, black, and white. These colors were selected based on previous studies of responses of dipteran pests, particularly tephritid flies, to traps of different colors [35,36,37]. A transparent Petri dish without any color was used as a control. A digital colorimeter (CR-20 plus, Konica Minolta, Ramsey, NJ, USA) was used to characterize each of the colors using the CIELAB color space system [38] (Supplementary Table S1). Each trap was filled with 30 mL of water and 10 µL of neutral detergent to reduce surface tension and facilitate fly capture. The nine traps were randomly arranged on the floor of a nylon cage (90 cm long × 60 cm wide × 60 cm high) in three rows, with 20 cm between rows and 20 cm between traps within each row. Cages had two LED lamps for homogeneous illumination of 1100–1200 lux in the upper part of the cage. For each replicate, 30 non-starved *S. adipata* adults (15 females and 15 males), aged 8–12 days, were released into the cage at 10:00 a.m. Trapped flies were collected from the Petri dishes 23 h later, sorted by sex, and counted. Flies remaining in the cage were discarded. A total of 15 replicates were conducted. Dishes were washed and randomly repositioned between replicates.

### 2.3. Visual Attraction to Different Colors in Fig Orchards

A field experiment was conducted in orchard 1 from 17 April to 15 May 2025 at the end of the dry season. The first rainfall during the experiment occurred on 5 May 2025 (Supplementary File S1). The orchard was divided into seven blocks, each measuring 250–300 m^2^. Transparent plastic bottle traps (500 mL) were constructed by drilling three equidistant holes (5 mm diameter) at two-thirds of the bottle height. The lower half of each bottle was spray-painted with one of three acrylic colors (Aero Comex, Mexico City, Mexico): (i) white, (ii) yellow, or (iii) orange (see CIELAB color characteristics presented in Supplementary Table S1). The colors used were selected based on the results of the laboratory experiments. An unpainted transparent bottle trap was used as a control. Each trap was filled with 250 mL of torula yeast suspension consisting of 1 pellet per 100 mL and placed on fig trees within each block at a height of 1.5–1.8 m above the ground. Traps on different trees were spaced 5–6 m apart, while blocks were separated by 8–10 m.

Traps were emptied at 7-day intervals, and collected flies were placed in 70% ethanol and transported to the laboratory for analysis. Traps were then topped-up with fresh torula yeast suspension to maintain the 250 mL volume and rotated to a new position within the block for the next 7-day period. The torula yeast suspension in each trap was replaced after two weeks. The experiment lasted for 4 consecutive weeks, ensuring that each trap was placed in each position once within its block.

In the laboratory, captured insects were counted and sorted by major orders: Diptera, Coleoptera, Hymenoptera, Lepidoptera, and other insects or arthropods. Lonchaeid flies were separated from other Diptera, counted, sexed, and grouped within the genera *Silba*, *Neosilba*, and *Lonchaea* using taxonomic keys [32,39].

### 2.4. Attraction to Odors Under Laboratory Conditions

#### 2.4.1. Comparison of Types of Attractants

A laboratory choice test was performed to compare attraction of *S. adipata* females and males to the following four attractants: (i) 2% (*w*/*v*) ammonium sulfate solution, (ii) Captor 300 + borax, (iii) torula yeast pellets (1 pellet per 100 mL), and (iv) CeraTrap. Small cup traps were constructed from 120 mL plastic cups (35 mm diameter, 87 mm height) that were perforated on the lateral wall with three equidistant holes at 45 mm above the base through which translucent conical tubes (9 mm external diameter, 6 mm internal diameter, 20 mm length) were inserted to decrease the frequency of fly escape once inside the trap [40]. The lower half of the cup was covered with cream-colored paper tape to facilitate fly landing on the surface of the trap and to avoid the influence of attractant color. Traps were loaded with 50 mL of each attractant and were randomly assigned to the corners of acrylic cages (30 × 30 × 30 cm) that were ventilated with 0.3 mm mesh on the lateral and upper sides of the cage. A moist cotton water source was placed at the center of the cage for the duration of each experiment. Sixteen non-starved unmated flies (eight females and eight males) of 8–12 d old were released inside the cage. Trapped flies were collected, sorted by sex, and counted at 23 h later. The remaining flies inside the cage were collected and discarded. The traps were rotated at each position within each cage to give a total of twenty replicates.

#### 2.4.2. Comparison of Ammonium Salts

Four separate pairwise laboratory choice tests were performed to compare *S. adipata* attraction to 2% ammonium sulfate solution with each of three concentrations of ammonium acetate solutions (2%, 0.2% and 0.02%, *w*/*v*). A comparison of 2% ammonium sulfate solution with distilled water was also included. Two plastic cup traps with a 120 mL capacity, one baited with 50 mL of 2% ammonium sulfate solution and the other with 50 mL of an ammonium acetate solution (0.02–2%), were placed on the opposite sides of 30 × 30 × 30 cm acrylic cages. Traps were initially randomly assigned to a particular size of each cage but subsequently changed in position for each new replicate. A moist cotton water source was placed at the center of the cage for the duration of each experiment. Ten non-starved unmated flies of *S. adipata* of 8–15 d old (five females and five males) were released inside the cage. Trapped flies were collected, sorted by sex, and counted at 23 h later. The remaining flies were collected and discarded. Four independent cages were evaluated simultaneously, and traps were evaluated at both positions in the four cages to give a total of eight experimental replicates.

### 2.5. Efficacy of Attractants in Fig Orchards

Field experiments were performed in orchard 1 between 16 May and 13 June 2024 (dry season) and in orchard 2 between 7 June and 4 July 2024, at the beginning of the rainy season. No rainfall was registered until 10 June 2024, after which periods of rainfall occurred 5–6 days a week (Supplementary File S1). According to data from a nearby climatological station located 4 km away, a total of 12.5 mm of rainfall was recorded during the experiment in orchard 1, whereas 515 mm was recorded during the experiment in orchard 2 (Supplementary File S1) [41]. Four attractants were evaluated in the field: (i) 2% ammonium sulfate solution, (ii) Captor + borax, (iii) torula yeast pellets (1 pellet per 100 mL), and (iv) 2% ammonium sulfate solution + 0.75 mL fig latex. These attractants were selected based on the results of the laboratory experiments. The mixture of ammonium sulfate + latex was included in the experiment due to the improved attraction to fig latex reported previously [31]. Fig latex was collected from exudates after removing green figs from the plants. The ammonium sulfate + latex mixture was prepared 10–15 min before hanging the traps on trees. The experimental orchards were divided into four blocks of 400 m^2^ each. Transparent plastic bottle traps (500 mL) were loaded with 250 mL of each of the four different attractants and placed on different fig trees at a height of 1.5–1.8 m above the ground. Traps on different trees were separated by 6–8 m, and blocks were separated by 10–12 m. Traps were emptied at weekly intervals, and flies were collected, placed in 70% ethanol, and transported to the laboratory. Traps were filled with fresh attractants and rotated one position within the block for the following week. The experiment was performed over four consecutive weeks, so that each trap was placed once at each position within each block.

The captured insects were counted and sorted into the orders Diptera, Coleoptera, Hymenoptera, Lepidoptera, and other arthropods. Lonchaeid flies were separated from other dipterans and classified as *Silba*, *Neosilba*, and *Lonchaea* using taxonomic keys [32,39]. Adults of *S. adipata* were sexed, and females were dissected under a stereomicroscope to determine the prevalence of sexually mature and immature females. Females were considered mature if at least one ovary contained mature oocytes [22].

### 2.6. Attraction to Ammonium Sulfate and Fig Latex in Fig Orchards

Based on the observed field attraction of *S. adipata* to 2% ammonium sulfate + fig latex, an additional experiment was conducted to evaluate the specific role of fig latex in the attraction. In this experiment, captures of *S. adipata* were compared between traps baited with 250 mL of water supplemented with 0.75 mL of fig latex and traps baited with 250 mL of 2% ammonium sulfate solution also supplemented with 0.75 mL of fig latex. Transparent plastic bottle traps (500 mL) were used in all treatments. Traps were placed on fig trees in four blocks at a height of 1.5–1.8 m above ground level. Within each block, traps were spaced 6–8 m apart, and blocks were separated by 10–12 m. Traps were emptied at weekly intervals, and captured flies were collected, preserved in 70% ethanol, and transported to the laboratory. After sampling, traps were refilled with fresh attractants and position within each block was changed for an additional one-week period (2 weeks in total). The experiment was conducted between 13 June and 27 June 2024 and repeated between 10 June and 24 June 2025. Captured *S. adipata* adults were counted and sexed but were not dissected.

### 2.7. Release of Ammonia from Traps

The release of gaseous ammonia from ammonium salt solutions was determined using an apparatus previously designed for this purpose [24,26]. The collection of ammonia was performed using a 5-liter opaque glass jar (255 mm height, 140 mm diam.) in which 50 mL of each attractant was placed in a 120 mL cup trap. The glass jar was sealed with a plastic lid that was perforated with two holes through which polytetrafluoroethylene (PTFE) tubes passed. Air was injected into the jar with the aid of an aquarium air pump (Elite 800, Rolf C Hagen UK, Castleford, UK). Injected air had previously passed through an activated charcoal filter and was regulated using a flowmeter (Cole-Parmer Scientific, Vernon Hills, IL, USA) at a rate of 150 mL/min. Another PTFE tube inserted into the other hole of the lid allowed air and volatiles to exit the jar and was connected to a 55 mL capacity glass tube (25 × 150 mm) containing 10 mL of distilled water to dissolve gaseous ammonia. Air was diffused into the water using an aquarium stone (Grupo Acuario de Lomas, Mexico City, Mexico) attached to the end of the tube as described previously [25,26]. The glass water trap was sealed with a perforated rubber bung through which a glass tube passed to allow the escape of exhaust gas. The dissolved ammonia in the distilled water trap was determined by measuring ammonium ion concentration through its reaction with Nessler reagent, quantified using an ammonia medium range photometer (Hanna Instruments Inc., Woonsocket, RI, USA). The Nessler reagent (K_2_HgI_4_) reacts with ammonium ions to produce a yellow-colored product, the intensity of which is directly proportional to the quantity of ammonia present. All measurements were performed in a climatically controlled laboratory at 24.5 ± 2 °C. The duration of the capture time of ammonia ranged from 2 to 5 h. The final quantity of ammonia was calculated for a 1 h capture time and expressed in micrograms NH_3_ per hour (µg/h) in four replicates per ammonia solution.

### 2.8. Statistical Analysis

The effects of color, attractant, and sex in the capture of *S. adipata* under laboratory conditions were analyzed by fitting generalized linear models (GLMs) with a quasi-Poisson error distribution to account for overdispersion. The numbers of *S. adipata*, *Neosilba* spp., and *Lonchaea* spp. captured per trap per week were used to fit GLMs with a binomial distribution or a quasi-Poisson error distribution, the results of which are presented as χ^2^ statistics. Means were compared by Bonferroni test. Pairwise comparisons of captures in traps containing ammonium sulfate solution or different concentrations of ammonium acetate solution or water were compared by paired *t*-test or Wilcoxon rank test for non-normally distributed data. The prevalence of captured females in field experiments and frequency of sexually immature females were analyzed by Fisher’s exact test. The rate of ammonia released from traps was compared by fitting GLMs. All analyses were performed using the R-based software Jamovi v. 2.3.28 [42].

## 3. Results

### 3.1. Visual Attraction to Different Colors: Laboratory Experiment

**Figure 2:**
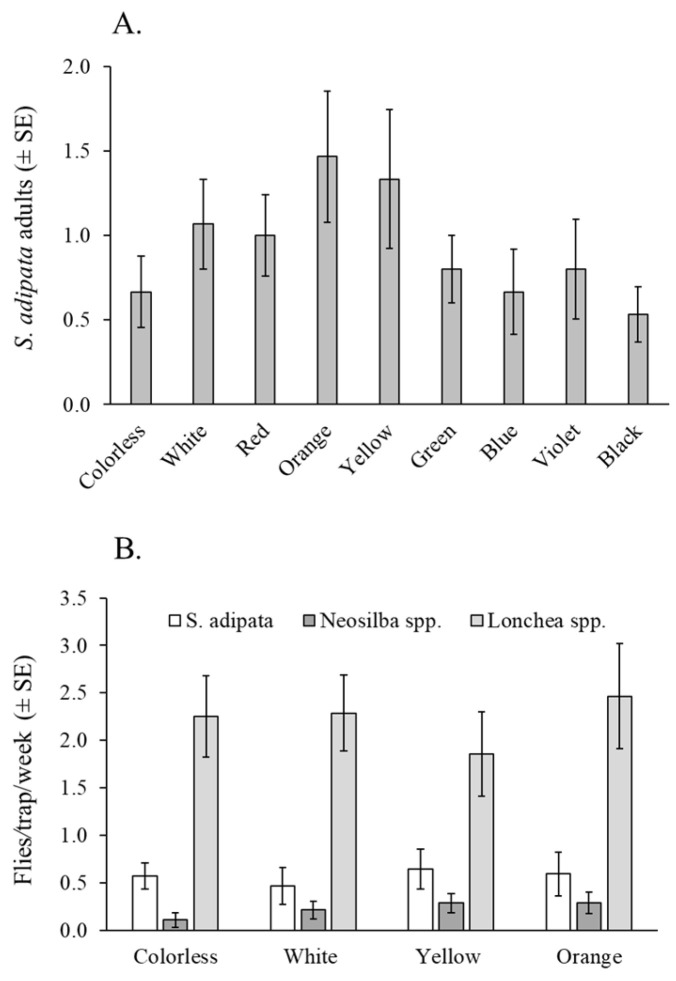
Fly responses to traps of different colors. (**A**) Mean *S. adipata* flies (both sexes) captured in colored water pan traps under laboratory cage conditions. (**B**) Mean flies per trap per week (both sexes) of *S. adipata*, *Neosilba* spp., and *Lonchaea* spp. in colored bottle traps baited with torula yeast pellets in a fig orchard.

A total of 125 *S. adipata* individuals, 81 females and 44 males, were captured using water pan traps in the laboratory. The mean number of flies captured did not differ significantly among trap colors (χ^2^ = 13.60, df = 8, *p* = 0.093), between sexes (χ^2^ = 1.47, df = 1, *p* = 0.225), or in the color × sex interaction (χ^2^ = 2.33, df = 7, *p* = 0.939) (Figure 2A).

### 3.2. Visual Attraction to Different Colors in Fig Orchards

A total of 305 lonchaeid flies were captured in this experiment, including 63 *S. adipata* adults (47 females and 16 males), 25 *Neosilba* spp. adults (22 females and 3 males), and 248 *Lonchaea* spp. adults (214 females and 34 males). The mean number of adults captured per week did not differ significantly among trap colors for *S. adipata* (χ^2^ = 4.60, df = 3, *p* = 0.204) or *Lonchaea* spp. (χ^2^ = 1.50, df = 3, *p* = 0.683). Due to the low number of *Neosilba* spp. adults captured (n = 25), this group was not subjected to formal statistical analysis. (Figure 2B).

The percentage of females trapped was highly variable among colors (mean 50–91%) but did not differ significantly across colors (χ^2^ = 4.18, df = 3, *p* = 0.243). Of the 47 *S. adipata* females captured, 25 (53%) were sexually immature, although the prevalence of sexual maturity did not vary significantly across different trap colors (χ^2^ = 0.0765, df = 3, *p* = 0.995).

In relation to the trap selectivity, all traps captured similar numbers of non-target insects (averaging 2.6 to 4.4 insects per trap per day), with no significant differences among colors (χ^2^ = 5.21, df = 3, *p* = 0.168). In this experiment, dipterans represented the vast majority of non-target insects captured, accounting for 98% of the total insects captured.

### 3.3. Attraction to Odors Under Laboratory Conditions

#### 3.3.1. Comparison of Types of Attractants

**Figure 3:**
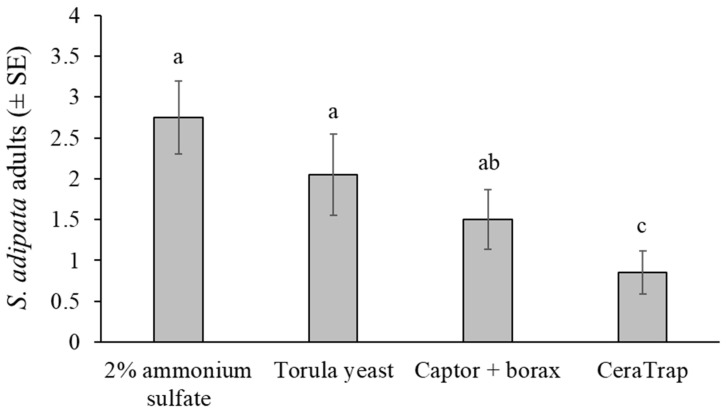
Mean *S. adipata* flies of both sexes captured in traps baited with different attractants in laboratory cage conditions. Columns labeled with different letters differed significantly (GLM, Bonferroni, *p* < 0.05).

A total of 143 *S. adipata* flies, 75 females and 68 males, were captured in cup traps across twenty replicates, representing 45% of the 320 flies released into cages. The mean number of flies captured differed significantly among attractants (χ^2^ = 19.108, df = 3, *p* < 0.001), but not between sexes (χ^2^ = 0.404, df = 1, *p* = 0.525) or in the attractant × sex interaction (χ^2^ = 0.313, df = 3, *p* = 0.957) (Figure 3).

Under laboratory cage conditions, adult *S. adipata* flies were captured in similar numbers by traps baited with the 2% ammonium sulfate solution and torula yeast pellets. Both attractants were significantly more effective than CeraTrap, whereas Captor + borax yielded intermediate capture rates.

#### 3.3.2. Comparison of Ammonium Salts

**Figure 4:**
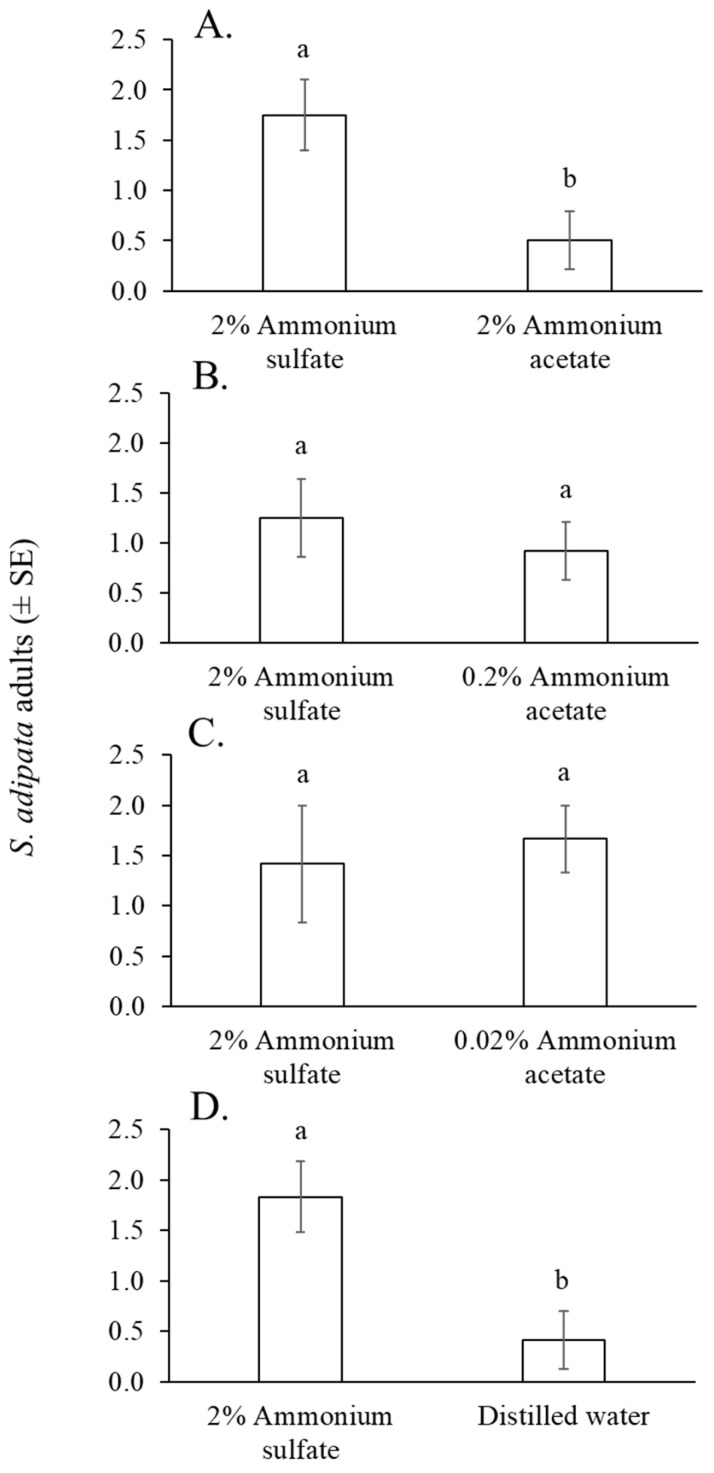
Mean number of *S. adipata* flies per trap per day captured in traps baited with solutions of 2% ammonium sulfate and 2% ammonium acetate (**A**), 0.2% ammonium acetate (**B**), 0.02% ammonium acetate (**C**), and distilled water (**D**). Columns labeled with different letters differed significantly (paired *t*-test, *p* < 0.05).

Between 27 and 37 *S. adipata* flies (55% females), were captured in cup traps across the eight cage replicates of the experiment, representing 33–46% of the 80 flies released. The mean number of flies captured in traps containing 2% ammonium sulfate solution was significantly higher than those captured with 2% ammonium acetate solution (paired t = 2.8, df = 11, *p* = 0.017) (Figure 4A), but not when compared with 0.2% ammonium acetate solution (paired t = 0.560, df = 11, *p* = 0.586) (Figure 4B) or 0.02% ammonium acetate solution (Wilcoxon W = 41, *p* = 0.502) (Figure 4C). A significantly higher number of flies was captured in traps baited with 2% ammonium acetate solution than those baited with distilled water (paired t = 2.41, df = 11, *p* = 0.034) (Figure 4D).

Ammonia release from solutions differed significantly among salts and concentrations (χ^2^ = 89.1, df = 3, *p* < 0.001). The 2% ammonium sulfate solution released a similar quantity of ammonia as the 2% ammonium acetate solution, and both released significantly higher levels of ammonia compared to solutions of 0.2% ammonium acetate or 0.02% ammonium acetate (Table 1).

### 3.4. Efficacy of Attractants in Fig Orchards

In orchard 1 at the end of the dry season, a total of 1106 lonchaeid flies were captured, comprising 622 *S. adipata*, 327 *Neosilba* spp., and 157 *Lonchaea* spp. Of the *S. adipata* adults, 377 (67%) were females and 185 (33%) males, while 60 individuals could not be sexed due to abdominal damage. In orchard 2 at the beginning of the rainy season, a total of 1080 lonchaeid flies were captured, including 488 *S. adipata*, 414 *Neosilba* spp., and 178 *Lonchaea* spp. Of the *S. adipata* adults, 246 (55%) were females and 199 (45%) males, with 43 individuals unsexed due to abdominal damage.

#### 3.4.1. *Silba adipata*

**Figure 5:**
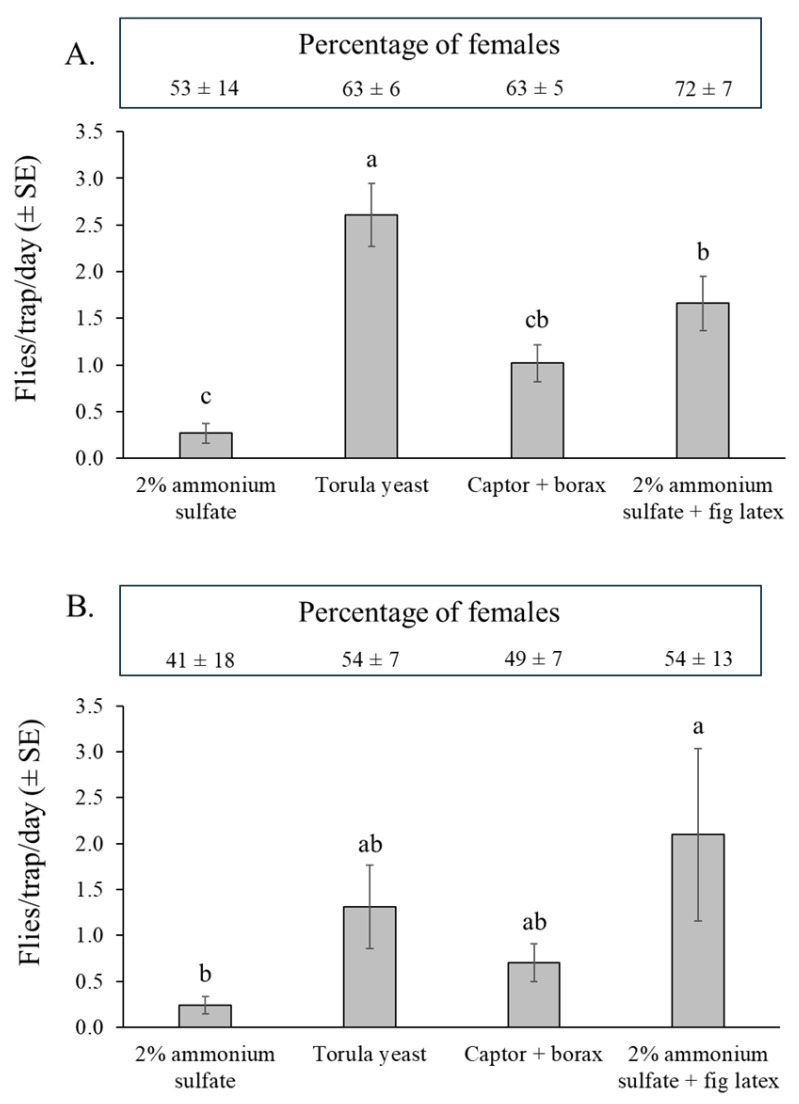
Mean (±SE) *S. adipata* flies per trap per day (FTD) captured in traps baited with different attractants in fig orchard 1 (dry season) (**A**), and fig orchard 2 (rainy season) (**B**). Columns labeled with different letters differ significantly (GLM, Bonferroni, *p* < 0.05). Boxes above graphs indicate the mean (±SE) percentages of females captured by each attractant in both experiments. The percentage of females captured did not vary significantly in either experiment (GLM, Bonferroni, *p* > 0.05).

In orchard 1 (dry season), torula yeast attracted a significantly higher number of *S. adipata* adults than any other attractant, followed by 2% ammonium sulfate + fig latex and Captor + borax, whereas captures in traps containing 2% ammonium sulfate solution were extremely low (χ^2^ = 65.4, df = 3, *p* < 0.001) (Figure 5A).

In the experiment at the beginning of the rainy season (orchard 2), captures were more variable than observed in the previous experiment (Figure 5B). The highest number of captures was registered in the traps containing 2% ammonium sulfate + fig latex and a significantly lower number of captures in the 2% ammonium sulfate solution treatment (χ^2^ = 12.7, df = 3, *p* = 0.005) and intermediate numbers captured in the traps containing Captor + borax and torula yeast pellets (Figure 5B).

Considering the 32 traps evaluated for each attractant across both experiments (128 trap samples in total), the percentage of traps with zero captures of *S. adipata* was significantly higher for 2% ammonium sulfate solution (60%) compared to the other three attractants, which had zero-captures in 12% of traps for torula yeast pellets, 19–22% in Captor + borax traps, and just 2% of traps containing ammonium sulfate + fig latex (χ^2^ = 21.3, df = 3, *p* < 0.001).

The percentages of *S. adipata* females captured (shown in boxes above Figure 5A,B), were similar among the different attractants in both orchard 1 (χ^2^ = 2.38, df = 3, *p* = 0.498) and orchard 2 (χ^2^ = 0.995, df = 3, *p* = 0.802). However, the overall sex ratio was significantly female-biased in the dry season experiment (orchard 1, 65 ± 4% females), but not in the rainy season experiment (orchard 2, 50 ± 4% females), so that the percentage of trapped females differed significantly between experiments (χ^2^ = 4.83, df = 1, *p* = 0.028).

The percentage of immature females did not vary significantly among attractants in either orchard 1 (62–85%) (χ^2^ = 2.22, df = 3, *p* = 0.529) or orchard 2 (79–100%) (χ^2^ = 3.66, df = 3, *p* = 0.301). The overall percentage of immature females was also similar between orchard 1 (mean 73 ± 4%) and orchard 2 (mean 89 ± 3%) (χ^2^ = 5.86, df = 1, *p* = 0.119).

A total of 103 out of a total of 1110 captured *S. adipata* adults could not be sexed from both experiments due to abdominal damage caused by degradation of the insect’s body in the drowning solution, an issue that may be important for monitoring purposes. Among the traps evaluated, the prevalence of unsexed *S. adipata* adults was significantly lower for Captor + borax (2%) and torula yeast pellets (5%) (χ^2^ = 70.1, df = 3, *p* < 0.001) compared to 2% ammonium sulfate + latex (16%) or 2% ammonium sulfate solution alone (27%), both of which tended to favor the decomposition of the trapped flies.

#### 3.4.2. *Neosilba* spp. and *Lonchaea* spp.

**Figure 6:**
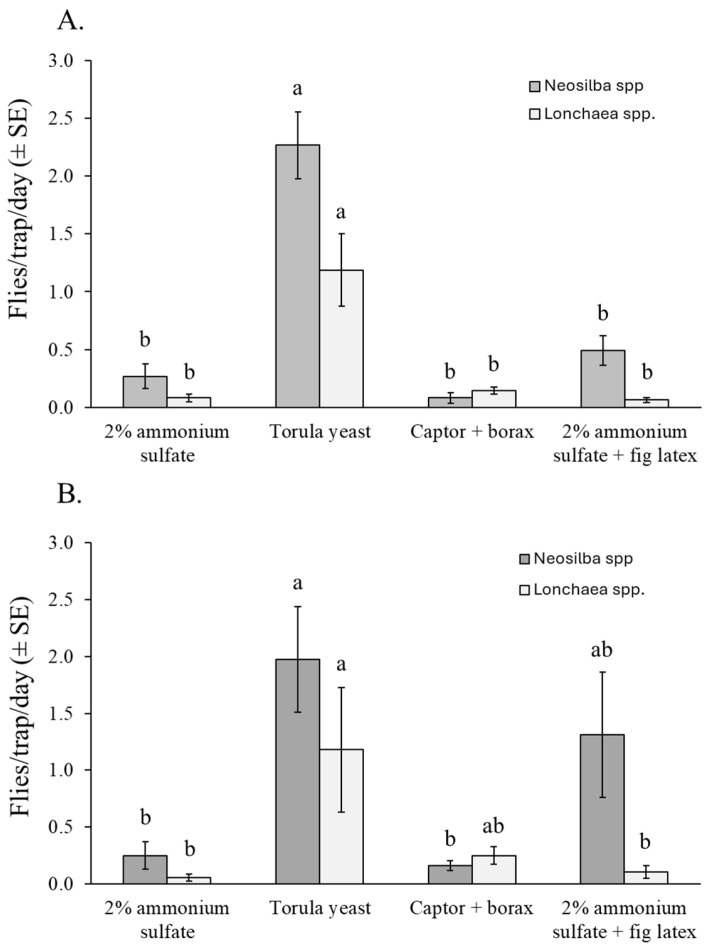
Mean captures of *Neosilba* spp. and *Lonchaea* spp. flies per trap per day (FTD) (both sexes) in traps baited with different attractants in fig orchard 1 (**A**) and fig orchard 2 (**B**). FTD values labeled with different letters differ significantly for *Neosilba* and *Lonchaea* (GLM, Bonferroni, *p* < 0.05).

Almost all the captured adults of *Neosilba* spp. (96%) and *Lonchaea* spp. (73%) were females, so no comparisons were made between the sexes. Torula yeast + borax was the most effective attractant for capturing adults of *Neosilba* spp. and *Lonchaea* spp., regardless of sex. However, differences were observed between experiments. The number of *Neosilba* spp. adults captured in torula yeast–baited traps was significantly higher than in traps baited with other attractants in the dry season in orchard 1 (χ^2^ = 106.57, df = 3, *p* < 0.001) (Figure 6A). However, in the rainy season in orchard 2, captures of *Neosilba* spp. were highest in the torula yeast treatment, lowest in the 2% ammonium sulfate solution and Captor + borax treatments, and intermediate in traps containing 2% ammonium sulfate + fig latex (χ^2^ = 14.306, df = 3, *p* = 0.030) (Figure 6B).

For *Lonchaea* spp., significantly more adults were captured in traps baited with torula yeast pellets than with any other attractant in orchard 1 (χ^2^ = 47.51, df = 3, *p* < 0.001) (Figure 6A), whereas in orchard 2, the number of *Lonchaea* spp. adults captured was significantly higher in the torula yeast baited traps than in ammonium sulfate treatments (χ^2^ = 9.18, df = 3, *p* = 0.027), although this did not differ significantly from the Captor + borax treatment (Figure 6B).

**Figure 7:**
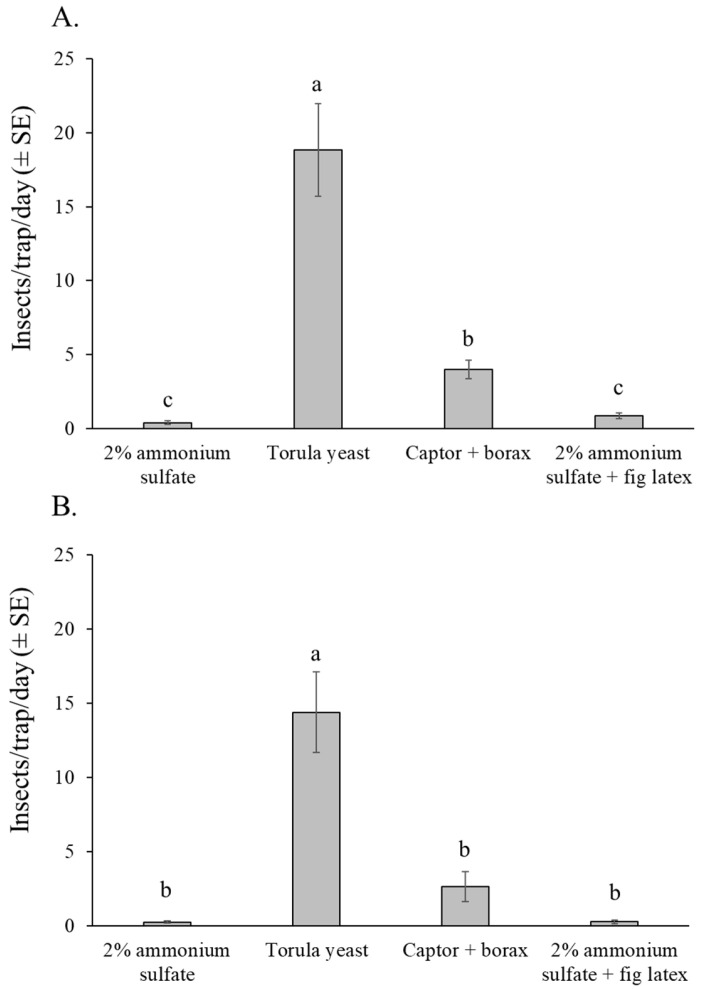
Mean number of insects per trap per day (excluding lonchaeid flies) captured in traps baited with different attractants in the fig orchard 1 (dry season) (**A**), and fig orchard 2 (rainy season) (**B**). Columns labeled with different letters differ significantly (GLM, Bonferroni, *p* < 0.05).

The selectivity of attractants in relation to non-target insect captures showed a similar pattern in both experiments (Figure 7A,B). The number of non-target insects was highest in traps baited with torula yeast pellets in both experiments (orchard 1: χ^2^ = 218.21, df = 3, *p* < 0.001; orchard 2: χ^2^ = 140.29, df = 3, *p* < 0.001). In orchard 1, Captor + borax captured significantly more non-target insects than either of the ammonium sulfate-based attractants (Figure 7A), whereas in orchard 2, the captor + borax and ammonium sulfate based attractants were similar in their captures of non-target insects (Figure 7B).

Most of the non-target insects captured in the traps belonged to the order Diptera (overall mean 81%), followed by Hymenoptera (overall mean 18%) (Supplementary Figure S1). Insects from the orders Lepidoptera, Coleoptera, and other insect orders, as well as other arthropods such as spiders, were rarely captured in traps and represented less than 1% of the overall captures. Most of the hymenopterans captured were ants, whereas beneficial insects such as pollinators or parasitoids were not captured in any of the traps.

### 3.5. Attraction to Ammonium Sulfate and Fig Latex in Fig Orchards

The traps baited with water + fig latex and monitored over the two-week evaluation period did not capture any *S. adipata* adults in June 2024 and only one male in June 2025. In contrast, eleven *S. adipata* adults (six females and five males) were captured in traps baited with 2% ammonium sulfate + fig latex in 2024 and seven adults (four males and three females) in 2025. Considering both years, the mean number of flies per trap per week was significantly higher for 2% ammonium sulfate + fig latex (1.1 ± 0.3 flies/trap/week) than for water + fig latex (0.06 ± 0.06 flies/trap/week) (Wilcoxon, W = 2.50, *p* = 0.020), indicating that latex enhanced fly captures only in the presence of the ammonium sulfate solution.

## 4. Discussion

The type of attractant and trap characteristics, such as color, are generally considered the most critical factors influencing trap performance [11,14]. Color attraction can vary among fly species and across different environments, with the contrast between the trap and its background playing a significant role in visual detection [43,44]. Despite improvements in tephritid captures using traps of different colors, no significant color preference was observed for *S. adipata* under laboratory conditions. Although yellow and orange had numerically higher but non-significant captures in the laboratory, these colors did not increase *S. adipata* captures in fig orchards compared to white or transparent bottle traps. In our field trials, no color preference was observed for any other lance flies, including *Neosilba* spp. and *Lonchaea* spp. Additionally, none of the trap colors demonstrated greater selectivity by reducing the capture of non-target insects. To our knowledge, this is the first study to directly evaluate the responses of lance flies to trap color, which greatly limits possible comparisons with previous studies. However, it is important to consider that recent insights into insect visual perception go beyond the human-centric interpretation of color, and include ultraviolet sensitive photoreceptors and the perception of polarized light that lie outside the human experience of the visual spectrum [45].

Ammonium sulfate was previously reported to be an effective attractant for monitoring *S. adipata* in Greece [8]. In our laboratory experiments the mean number of flies captured with 2% ammonium sulfate solution was significantly higher than with 2% ammonium acetate solution (Figure 4), despite both releasing nearly equivalent amounts of ammonia (Table 1). However, when ammonium acetate was used at lower concentrations (0.2% or 0.02%), it performed similarly to 2% ammonium sulfate solution. We hypothesize that at 2%, acetate ions may act as weak bases, capturing protons from water and generating small amounts of volatile acetic acid, which could be repellent to *S. adipata*, although further experiments are clearly required to confirm this hypothesis. Incidentally, tephritids avoid acidic environments, as they do not associate them with viable food or protein sources [46].

The values of ammonia released by traps containing ammonium salt solutions (0.3–3.1 µg/h, Table 1) were broadly similar to that of torula yeast pellets (1.8 ± 0.4 µg/h) but notably lower than the tephritid attractant CeraTrap (11.5 ± 3.5 µg/h) or the hydrolyzed protein product Captor + borax (20.4 ± 2.7 µg/h) that were previously measured under the same conditions [26,34]. In any case, despite its widespread use in tephritid monitoring [23], ammonium acetate was no more effective than ammonium sulfate in attracting *S. adipata*.

Significant differences were observed in the capture of *S. adipata* adults between the laboratory and fig orchard experiments. The odor-neutral conditions of the 24 h laboratory assays differed greatly from the conditions in the field trials, in which attractant performance is subjected to the influence of climatic conditions and competes with the complex blend of volatiles naturally released by fig plants. Although the 2% ammonium sulfate solution was effective in attracting *S. adipata* under laboratory cage conditions, it failed to do so in the field probably due to interference from the presence of fig volatiles, yielding the lowest number of captures, the highest frequency of traps with zero captures, and the highest percentage of adults with abdominal degradation that prevented them from being sexed. In contrast, torula yeast pellets captured a greater number of *S. adipata* adults in both field experiments, although significant differences compared to the 2% ammonium sulfate solution were only observed in the dry season experiment (orchard 1). The attractiveness of torula yeast likely increases over time as a result of microbial decomposition, a process influenced by temperature, as described below. In the field, the combination of 2% ammonium sulfate + fig latex was also attractive to *S. adipata* and captured significantly more adults than the 2% ammonium sulfate treatment. Captor + borax generally attracted intermediate numbers of *S. adipata* adults, and capture rates for this attractant were lower than those observed for either torula yeast pellets or the ammonium sulfate + fig latex mixture. The commercial tephritid attractant CeraTrap performed poorly and does not appear to be a useful attractant for *S. adipata*.

The performance of torula yeast pellets differed between the two field experiments, likely due to varying weather conditions. Torula yeast was more attractive to *S. adipata* during the dry season experiment in orchard 1 than during the rainy season experiment in orchard 2. This difference may be attributed to the lower mean temperatures observed during the second experiment, caused by increased rainfall, and the known delayed release of ammonia from torula yeast pellets, which typically require several days to reach effective levels of ammonia emission [34]. Elevated temperatures during the dry season may have accelerated microbial decomposition of the torula yeast pellets, thereby enhancing the release of ammonia and other protein-derived volatiles, ultimately increasing the efficacy of the attractant as has been observed in *Anastrepha obliqua* [47].

The spacing of traps in field studies was 5–6 m, which may initially appear rather close raising the question of interference between adjacent traps from different treatments. There is very limited published information on optimal trap spacing for lonchaeid pests. Nonetheless, potential interference or competition among traps is not expected to have significantly influenced our results, mainly because the fig trees at the study site were planted at intervals of 2 m so that there were 2 or 3 trees between each trap—reducing the likelihood of interference, although clarification of this issue requires systematic study.

A higher prevalence of females (55–65%) was captured in field experiments compared to males, although no significant differences were observed in the percentages of females among the different attractants. Females of *S. adipata* are probably more active in seeking protein sources than males, driven by the requirement for protein in ovarian maturation [22]. This pattern mirrors the behavior observed in tephritid flies, which also rely on dietary protein for reproductive development [48,49]. Female-biased responses to protein-based attractants have been previously reported for *S. adipata* using ammonium sulfate or hexanol [28], as well as in several species of tephritid flies across different protein attractants [21].

The high attraction of females is particularly advantageous for reducing crop infestations, as females are responsible for oviposition. Interestingly, our results also revealed that a high prevalence of field-captured females were sexually immature (overall 78% of females), although long-term sampling across several generations or across a complete fig production cycle would be required to validate this finding. Under laboratory conditions, females of *S. adipata* consume hydrolyzed protein over a prolonged time (1–3 weeks) to achieve sexual maturity [22]. This extended maturation period could potentially explain the high fraction of sexually immature females in field populations. This finding is significant, as attractants such as torula yeast pellets or ammonium sulfate combined with fig latex, which captured 76–90% immature females, could be used strategically for mass trapping of the pest prior to the oviposition period, thus reducing fruit damage and infestation.

Few studies have evaluated the efficacy of attractants for *S. adipata*, and none have included hydrolyzed proteins or torula yeast pellets. In contrast, dozens of studies over the past five decades have assessed the efficacy of hydrolyzed proteins and ammonium salts as attractants for tephritid flies, revealing species-specific differences in attraction responses [21]. In a previous study conducted in the state of Morelos, Mexico, 2% and 4% ammonium sulfate solutions had limited effectiveness for monitoring *S. adipata*, followed by diammonium phosphate, whereas hexanol alone or in combination with other compounds was found to be ineffective [28].

Our findings indicate that torula yeast pellets or a mixture of 2% ammonium sulfate plus fig latex are likely to be effective attractants. However, for monitoring purposes, torula yeast + borax presents a major drawback in that it is highly attractive to other lonchaeid species (e.g., *Neosilba* and *Lonchaea*), and to non-target dipterans and hymenopterans (mostly ants). This reduces the specificity of the trap and markedly increases the time required for accurate pest identification when examining the trapped insect material. Due to the high morphological similarity among lonchaeid species, the capture of non-target lonchaeids could be a source of false positives by inexperienced growers [29]. Although not identified in the present study, *Lonchaea cristula* and several other species of *Neosilba*, including *N. batesi*, *N. glaberrima*, and *N. recurva* have been reared from figs collected at our field sites in the state of Veracruz [7,50].

It is important to note that the field experiments described here were all short-term and will have been influenced by the pest population density, crop phenology, and the climatic conditions experienced during the experimental period. The validation of our results will require longer term field trials that encompass multiple pest generations spanning the complete crop production cycle and across wet and dry seasons in this region. For example, the field trial testing the combination of ammonium sulfate and fig latex had low captures in both 2024 and 2025, so the results should be viewed with caution. This experiment was performed at the beginning of the rainy season in June of both years when levels of precipitation typically increase more than three-fold compared to the preceding month of May [51]. The rainy season is also marked by tropical depressions which may have affected fly activity, as we have observed a notable decrease in fly activity during periods of low atmospheric pressure, as also reported for other dipteran species [52,53]. This emphasizes the value of verifying our findings in future field trials over longer time spans.

The combination of 2% ammonium sulfate and fig latex was not only an effective attractant, but it was also significantly more selective than torula yeast pellets, attracting fewer non-target insects and lonchaeid flies. These are valuable characteristics for monitoring *S. adipata*. However, a key limitation is that a fraction (16%) of the captured adults could not be sexed due to abdominal degradation. Given that fig latex can be easily collected by growers, its use holds substantial potential for pest management due to its demonstrated effectiveness and selectivity. Fig latex is a complex substance comprising a wide variety of phytochemicals and antioxidants that release an elaborate volatile profile [54,55,56]. Future research should evaluate the attractant efficacy of the main volatiles present in fig latex or those generated in combination with ammonium sulfate, as well as alternative mixtures containing fig latex.

## 5. Conclusions

This study demonstrates the effectiveness of torula yeast pellets and mixtures of ammonium sulfate and fig latex for capturing *S. adipata* in traps. These findings should contribute to monitoring the establishment and spread of this invasive pest in Mexico and elsewhere, but merit validation in future field studies performed over the complete crop cycle and across different seasons. Additional studies should be conducted to evaluate other proteins or ammonium salt combinations in order to develop more effective and selective monitoring or mass trapping tools to manage this exotic pest.

## Figures and Tables

**Table 1:** Quantity of gaseous ammonia released by traps over a one-hour period for the different ammonium salts at various concentrations.

Attractants	µg/trap/h
2% ammonium sulfate	3.1 ± 0.4 a
2% ammonium acetate	3.1 ± 0.3 a
0.2% ammonium acetate	0.9 ± 0.2 b
0.02% ammonium acetate	0.3 ± 0.1 b

Mean values followed by different letters differed significantly (GLM, Bonferroni *p* < 0.05).

## Data Availability

The original contributions presented in this study are included in the article and Supplementary Material. Further inquiries can be directed to the corresponding authors.

## Supplementary Materials

The following supporting information can be downloaded at https://www.mdpi.com/article/10.3390/insects16070732/s1, Figure S1: Mean (±SE) number of insects of the orders Diptera and Hymenoptera captured per trap for the different attractants during the two field experiments in orchard 1 and orchard 2. File S1: Daily precipitation during the periods of two field experiments in orchard 1 and orchard 2. Table S1: Mean color space of L*a*b* parameters for (A) adhesive plastic circles used in a laboratory cage experiment and (B) acrylic paint applied to field traps. File S2. Raw data presented in the study.
