# Supplementary material for: Color and Attractant Preferences of the Black Fig Fly, Silba adipata: Implications for Monitoring and Mass Trapping of This Invasive Pest
Source: Insects. 2025 Jul 17;16(7):732. doi: 10.3390/insects16070732 (PMC12295857; doi:10.3390/insects16070732)

Figure S1. Mean (± SE) number of insects of the orders Diptera and Hymenoptera captured per trap for the different attractants during the two field experiments in orchard 1 and orchard 2. Diptera and Hymenoptera (ants) comprised 99% of the insects captured.

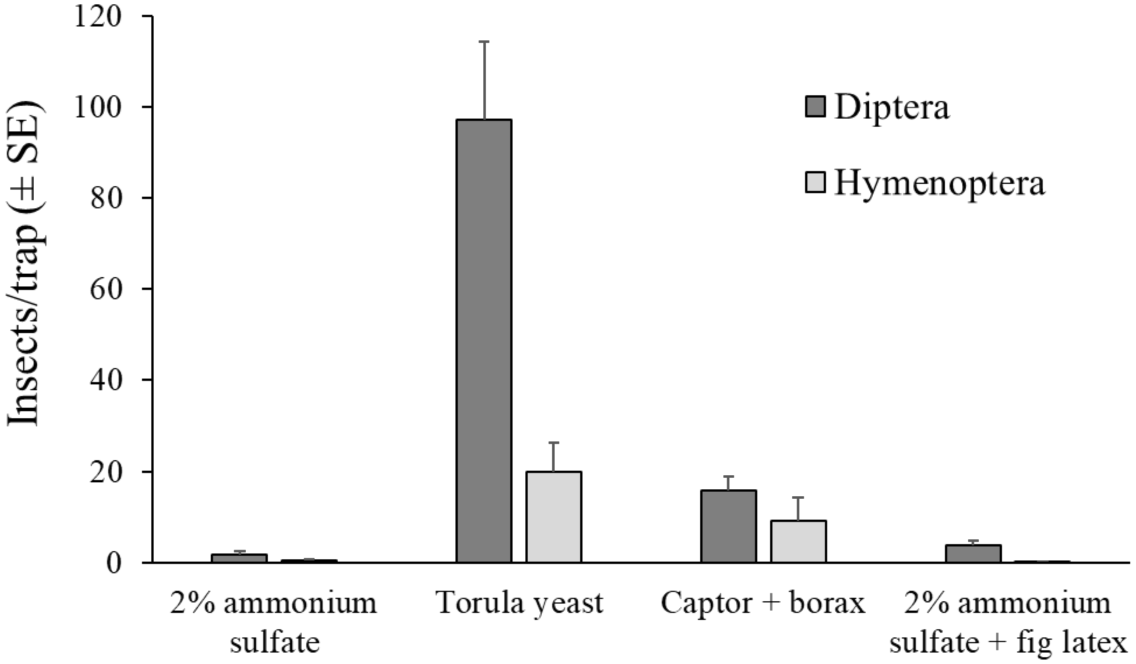

Supplement: Supplementary file 1 [file insects-16-00732-s001.zip › Figure S1.pdf]
