# Supplementary material for: Color and Attractant Preferences of the Black Fig Fly, Silba adipata: Implications for Monitoring and Mass Trapping of This Invasive Pest
Source: Insects. 2025 Jul 17;16(7):732. doi: 10.3390/insects16070732 (PMC12295857; doi:10.3390/insects16070732)
Supplement: Supplementary file 1 [file insects-16-00732-s001.zip › Table S1.pdf]

**Table S1**. Mean color space of L*a*b* parameters for A) Adhesive plastic circles used in a laboratory cage experiment and B) Acrylic paint applied to field traps. L* (lightness), a* (green to red), and b* (blue to yellow).

| A) Adhesive circles | L* | a* | b* |
|---|---|---|---|
| White | 87.0 | -2.1 | -4.7 |
| Red | 40.2 | 47.1 | 22.5 |
| Orange | 61.2 | 30.6 | 37.2 |
| Yellow | 71.0 | 10.9 | 55.2 |
| Green | 47.3 | -36.3 | 17.7 |
| Blue | 51.9 | -22.4 | -32.2 |
| Violet | 36.4 | 14.9 | -41.9 |
| Black | 26.4 | -0.6 | -1.8 |

| B) Acrylic paint | L* | a* | b* |
|---|---|---|---|
| White | 91.6 | -1.4 | -1.2 |
| Orange | 45.4 | 39.7 | 34.1 |
| Yellow | 73.7 | 4.3 | 71.7 |

Each value represents the average of four measurements.
